# Behavioural patterns of university students during the COVID-19 pandemic: A cross-sectional study of the effects of active transportation, uninterrupted sitting time, and screen use on physical activity and sitting time

**DOI:** 10.12688/f1000research.117843.3

**Published:** 2025-01-29

**Authors:** Gonzalo Marchant, Guillaume Chevance, Andrés Ladino, Brice Lefèvre, Nicolas Jacquemond

**Affiliations:** 1 Center for the Study and the Transformation of Physical Activities UR 3832, Faculty of Sport Sciences, University of Rouen Normandy, Mont-Saint-Aignan, Normandy, 76130, France; 2 Barcelona Institute for Global Health, ISGlobal Barcelona, Barcelona, 08003, Spain; 3 ENTPE, LICIT UMR-T9401, Gustave Eiffel University, Lyon, 69500, France; 4 L-ViS. F-69622, Université Claude Bernard Lyon 1, Villeurbanne, 69100, France; 5 University Service of Physical and Sports Activities (SUAPS), Université Claude Bernard Lyon 1, Villeurbanne, 69100, France

**Keywords:** Universities, sitting position, transportation, lifestyle, young people

## Abstract

**Background:**

The closure of universities due to the coronavirus disease 2019 (COVID-19) pandemic significantly affected students’ behaviours, particularly regarding physical activity, sitting time, and screen use. This study aimed to determine the effect of active transportation duration, uninterrupted sitting time, and screen time to study on physical activity and sitting time during the confinement.

**Methods:**

This was a cross-sectional study based on data collected via an online questionnaire for university students during the second confinement in France (between October and December 2020). The questionnaire assessed physical activity and sedentary behaviour, and contained questions about modes of transport, and perception of uninterrupted sitting time and screen time to study prior to confinement and during confinement. Participants (N=2873) completed the International Physical Activity Questionnaire (IPAQ) in an average time of around 15 minutes, after providing digital informed consent. Multiple regression models assessed how time duration of active transportation, uninterrupted sitting time, and screen time studying increased or reduced confinement effects on physical activity and sitting time.

**Results:**

The regression models showed that physical activity decreased during confinement for students who engaged in more prolonged periods of active transportation prior to confinement. Moreover, the perception of long, uninterrupted sitting time and high screen time prior to confinement significantly increased sitting time during confinement. Students who adopted the most active transport time prior to confinement were the least likely to increase their screen time during confinement.

**Conclusions:**

Confinement reduced physical activity levels and increased sitting time, mainly among students who adopted active transport and accumulated longer uninterrupted sitting time prior to confinement. Students who combined-long periods of uninterrupted sitting time with high screen use could be a riskier profile for health. Analysis of physical activity time and sitting position should include its accumulation patterns.

## Introduction

The confinement caused by the coronavirus disease 2019 (COVID-19) pandemic significantly reduced the physical activity levels of university students. ^1^ This effect was primarily due to the reduction in active transportation for commuting ^2^ and the subsequent increase in sitting time and screen time. ^3^ However, the decrease in physical activity levels was more pronounced for individuals who were more active before COVID-19, men, ^4^ and younger adults (ages 18-34). In contrast, older adults (>34 years old) experienced no significant changes in their physical activity levels. ^5^

Adopting active transportation, which includes any human-powered forms of travel, such as walking or cycling, makes people achieve the recommended physical activity levels for health (i.e., 150 minutes/week moderate physical activity). ^6^ Students who adopt active transportation display higher physical activity levels than those who do not adopt it. ^7^^–^^9^ Similarly, people who adopt longer time of active transport increase their total physical activity levels more than those who engage in shorter time of this activity. ^10^ Furthermore, active transport, such as walking, is a powerful preventive measure against physical conditions such as excessive weight, ^11^ type 2 diabetes, ^12^ and cardiovascular diseases. ^13^ It also reduces the likelihood of suffering mental issues such as stress, anxiety, depression, and cognitive impairments. ^14^

Although the threshold for physical activity to achieve health benefits is well defined, this is not the case for sitting time. ^15^ Despite this lack of evidence, recent studies suggest that uninterrupted sitting for periods longer than 30 minutes has a detrimental impact on health. ^16^ A threshold of six to eight hours per day of sitting and three to four hours of television is a health risk. ^17^ Students spend more than six hours per day studying while sitting down, ^18^ thus increasing health risks when sitting time accumulates uninterrupted. ^19^ Besides this sitting time, students increasingly use screens to study sitting, ^20^ mainly computers. ^21^ Another study suggested that computer use was a prevalent sedentary behaviour in university students, resulting in prolonged periods of sitting and, consequently, greater sedentary time. ^22^

While there has been a tendency to associate screens with sitting time, screen use does not always reflect that behaviour. ^23^ Regardless of sitting time, screen time could be an independent health risk factor. ^24^ Furthermore, the relationship between physical activity, sitting time and screen time is mixed. A systematic review showed no association between physical activity and sitting time. ^25^ Even high physical activity levels do not mitigate the adverse effects of high screen time on health. ^26^ Some studies have shown that students’ physical activity levels are negatively associated with sitting time, ^27^ where high sitting time displaces total physical activity time, ^28^ mainly light physical activity. ^29^ Similarly, when people spend more than six hours sitting, they replace that time with walking and moderate to vigorous physical activity. ^30^

According to Heller and colleagues, ^31^ sitting time during the COVID-19 pandemic was significantly lower among students who were highly physically active before the pandemic than among physically inactive students. The same authors showed no differences in sitting time between students who were moderately physically active and those who were physically inactive. In the same study, there were no differences between students who were highly physically active and those who were only moderately physically active.

In the literature related to the COVID-19 pandemic, university students would not show significant changes in their screen time related to sedentary time or physical activity, as students with higher levels of sedentary time also had higher levels of screen time. ^31^^,^^32^

Most studies about confinement in the COVID-19 pandemic have focused on total times of changes in physical activity levels and sitting time ^33^ or screen time. ^34^ However, it is suggested that not only the total duration of these behaviours, but also the way it is accumulated throughout the day (e.g., in a few long periods of time or in several shorter periods of time) might have a different effect on health. ^35^^,^^36^ Furthermore, more research needs to study the accumulation of these times before and during COVID-19 pandemic in university students. To our knowledge, no studies have explored active transportation duration, uninterrupted sedentary time, and the time using screens for studying before and during the COVID-19 pandemic.

**Figure 1.**
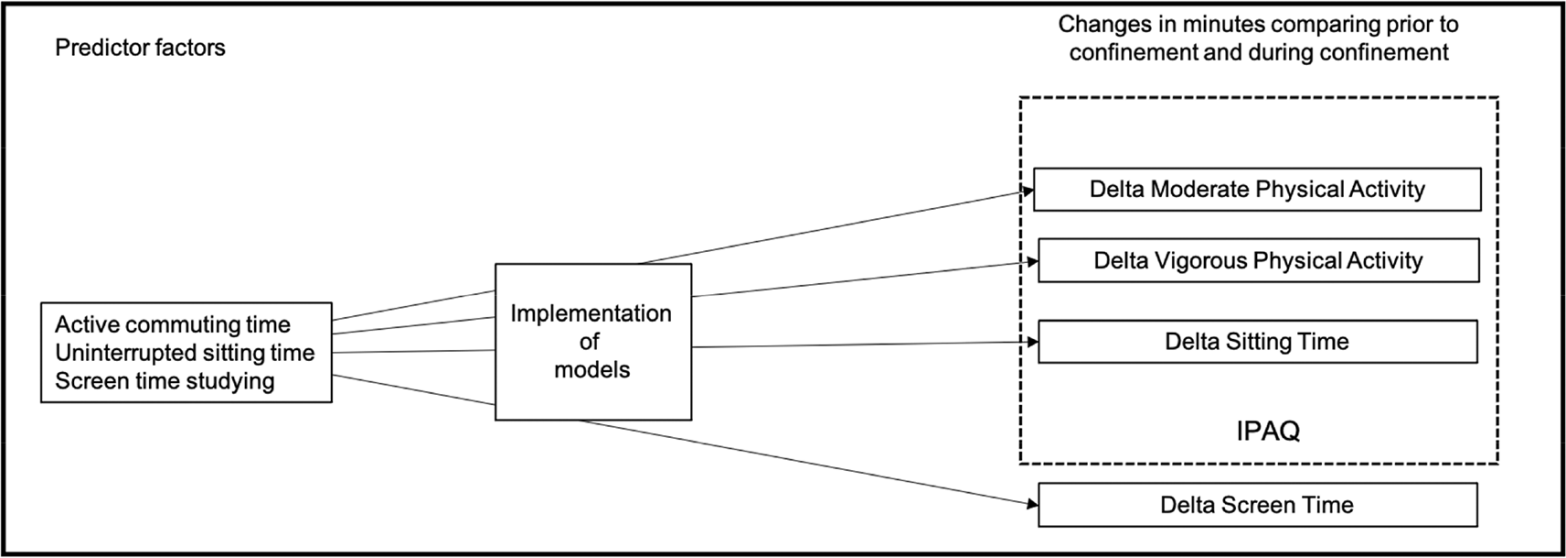
Schematic overview of the study objectives. IPAQ=International Physical Activity Questionnaire.

For this reason, the main objective of this study was to assess whether the duration of active transportation time, uninterrupted sitting time, and screen time spent studying before confinement determinate physical activity, sitting and screen times during confinement ( Figure 1).

### Hypotheses

*H1*: The first hypothesis was that the longer the duration in minutes of students’ active transportation trips prior to confinement, the greater the reduction in their physical activity levels, excluding active transportation time during confinement.

*H2*: The second hypothesis was that the greater the uninterrupted sitting time prior to confinement in students, the greater the increase in the amount of time they would spend sitting during confinement.

*H3*: The third hypothesis was for screen time; due to the increase in online courses during confinement, the longer the screen time studying prior to confinement in students, the greater the increase in their screen time during confinement.

*H4*: Finally, we hypothesised that there would be no relationship between active transportation, sitting time and screen time during confinement.

## Methods

**Figure 2.**
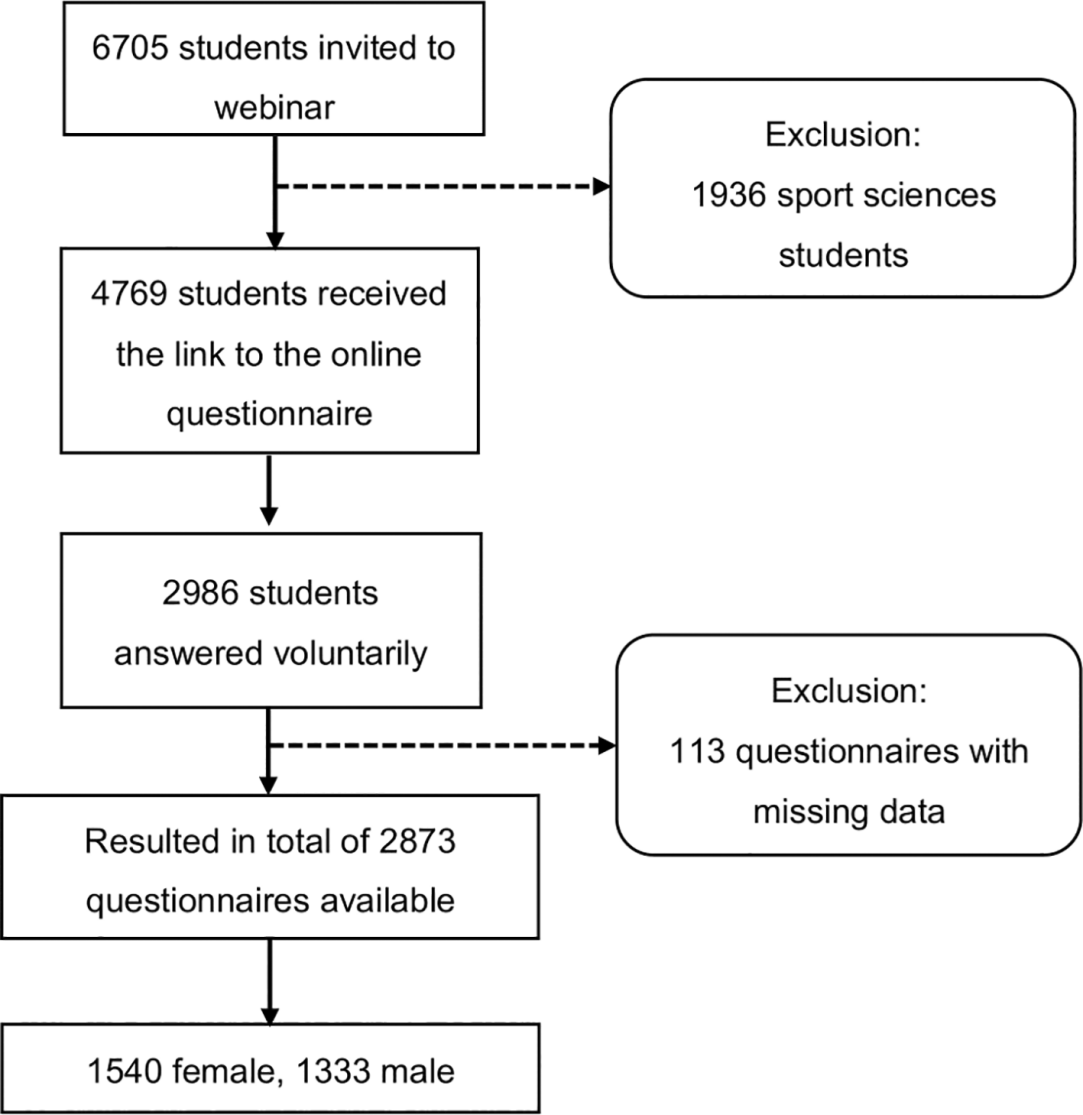
Flowchart of the study.

This was a cross-sectional study. Students from 16 faculties enrolled (N=6705) in the university sports service (SUAPS) received an invitation via the institutional e-mails to a health webinar for French students (Webex ^®^) (November 10, 2020) on physical (in) activity and sedentary behaviours during the confinement. A link to an online questionnaire was then sent to each student via the institutional e-mail addresses 15 minutes after the end of the Webinar, except for sports sciences students. Due to the type of training, these students were the only ones who partially maintained physical activities during the confinement, which would bias the data. The questionnaire was a voluntary survey, and it was available on the Drag n Survey ^®^ platform between November 10th and 18th, 2020. The survey used in this study is available as *Extended data.* ^37^ There was a separate informed consent page where students agreed to participate by checking a checkbox. The survey invitation was sent twice to increase response rates, and participants were offered access to the final research results as a non-monetary incentive. A total of 2986 students visited the website and agreed to participate in the study and only 2873 completed the screening questionnaire. Each questionnaire included a completeness check with forced response items ( *i.e.*, highlight mandatory items). In order to prevent a single user from filling in the same questionnaire multiple times, one response per IP address was possible (the flowchart is shown in Figure 2).

### Ethics

The National Commission for Data Protection and Liberties (CNIL-France) approved the study (2221060v0-CNIL). The final decision was determined on 8 ^th^ February 2021. The posteriori approval of our study is because it was not possible to know that the second wave of COVID-19 would occur in France. The protocol was submitted one week before the second COVID wave in France and initially this study was only supposed to investigate the effects of the first wave of COVID-19. We then adapted the protocol to examine the condition of confinement in the second wave of the pandemic.

The statement followed the Declaration of Helsinki and the French methodological reference MR-01. Students who visited the informed consent page and agreed to participate were considered to have provided informed consent. If participants did not want to participate in the survey, they could turn off the electronic questionnaire and drop out.

### Measures

*Demographics.* Information was collected, including sex and age in years (open-ended question).

*Physical activity.* Participants answered questions about how much time and how many days they spent doing moderate and vigorous physical activities during one week (excluding the weekend): First, “prior to the confinement” and then “during the confinement”. The survey was based on the International Physical Activity Questionnaire (IPAQ) short form. ^38^

*Transportation type for commuting.* Participants were asked what type of transport they habitually used to commute to university (open-ended question). Following which, the participants answered the following question: “On a typical day (prior to confinement), how many minutes do you spend carrying out active transportation?”. A definition of active transportation was then proposed to the participants in the following statement: “Active transportation is defined as any form of human-powered transportation ( *e.g.*, walking and cycling)”.

*Sitting time.* The IPAQ short form included the following question: “How much time did you spend sitting during a weekday? ( *i.e.*, hours and minutes per day, excluding the weekend)”. For this study, this question was asked prior to confinement and during confinement.

*Uninterrupted sitting time.* Students reported the average time of uninterrupted sitting. The question was, “On average (prior to confinement), how long do you sit without getting up? (hours and minutes)”.

*Screen time.* Students indicated average minutes per day of screen-time usage prior to and during confinement. The question posed with regards to their time prior to the confinement was: “Normally (prior to confinement), how many hours on average per day do you spend in front of a screen (computer, tablet, smartphone, TV…)?”. The question posed with regards to their time during the confinement was: “How many hours on average per day (during confinement) do you spend in front of a screen (computer, tablet, smartphone, TV)?”.

*Screen time studying.* Students reported their average daily hours using a computer ( *i.e.*, desktop, laptop, tablet) or smartphone to study. The question posed was, “Normally (prior to confinement), how many hours on average per day do you spend in front of a screen studying (computer, tablet, smartphone)?”. The question posed with regards to their time during the confinement was: “Normally (during confinement), how many hours on average per day do you spend in front of a screen studying (computer, tablet, smartphone)?”.

### Statistical analysis

We calculated the differences ( *i.e.*, delta Δ) in physical activity, sitting and screen time between the period prior to confinement and the period during confinement, using Microsoft Excel version 16.48 (RRID:SCR_016137). These differences were the dependent variables for four multiple regression models. Sex and age were included in the regression models as control variables to assess the impact of confounding variables. A graphic method of generalized additive models (GAM) ^39^ examined subgroups and interactions. The graphs are available as *Extended data.* ^40^ Missing data were handled by excluding incomplete cases from the dataset, avoiding the use of multiple imputations.

The models were used to prove our fourth hypothesis. The first regression model was for moderate physical activity and the second regression model was for vigorous physical activity. Both models were used to test the first hypothesis, which stated that the longer time a student adopted active transportation, the greater the reduction in physical activity minutes during confinement. The third model tested the second hypothesis that those who spent longer sitting for an uninterrupted time prior to confinement, the greater the increase in sitting time during confinement. The fourth model tested the third hypothesis, which suggested that the higher time students spent in front of a screen for study, the greater the increase in screen time during confinement. The four models tested our fourth hypothesis about the relationship between active transportation, sitting time and screen time during confinement. All regression analyses were performed with RStudio (2021.09.0) (RRID:SCR_000432) and the package lme4 (RRID:SCR_015654). ^41^

## Results

Table 1 presents the characteristics of 2873 students. ^42^ Overall, the sample was balanced between men (46%) and women (54%). They were aged between 18 and 29 years old (M=19.93, SD=1.76) and they were, according to IPAQ classification, inactive prior to confinement (60%). Students adopted walking (70%) as a mode of active commuting, 17% participated in cycling, 8% were passive commuters ( *e.g.*, car, bus, underground), 3% combined walking and cycling, and 2% adopted any combination of active and passive transport ( *e.g.*, bus, tramway and walking or tramway and cycling). The average duration of active transportation, prior to confinement, was approximately 30 minutes per trip. Moreover, the students spent approximately eight hours sitting down per day prior to confinement and with an average uninterrupted sitting time of almost 100 minutes prior to confinement. Students spent more than 10 hours per day using screens during confinement, of which approximately five hours were for studying and five hours outside of study hours. Analyses of the GAM models did not show the presence of subgroups within the data or interactions between variables. ^40^

**Table 1.** Descriptive statistics.

Variable	n
Sex	
Women	1540
Men	1333
Age, years, mean (SD)	19.93 (1.76)
18	488
19	916
20	693
21	387
22	199
≥23	202
IPAQ categories, before confinement	
Low	1738
Moderate	523
High	612
IPAQ categories, during confinement	
Low	1932
Moderate	448
High	493
Time of commuting to university in minutes per trip, mean (SD)	28.61 (18.44)
Passive commuting (bus, car and underground)	229
Active transportation time categories	
Between 10-15 min	447
>15-30 min	723
>30-45 min	1025
>45-60 min	337
>60 min	112
Uninterrupted categories of sitting time in minutes, mean (SD)	97.53 (63.00)
Sitting time categories	
15-30 min	259
>30-45 min	327
>45-60 min	695
>60 min	1592

IPAQ=International Physical Activity Questionnaire.

### Hypothesis

Four multiple regression models assessed the extent to which active transportation, uninterrupted sitting time, and screen time studying before confinement exacerbated or attenuated the effects of confinement on total physical activity time and sitting time. Multiple regression models indicate that when the variables had a negative effect, there was significantly less time reduction during the confinement. Conversely, when the effect was positive, it predicted a more considerable time change during confinement.

*H1*: The first hypothesis was that the longer the duration in minutes of students’ active transportation trips prior to confinement, the greater the reduction in their physical activity time during confinement.

To evaluate our first hypothesis, we conducted two multiple regression analyses ( Table 2), one for moderate physical activity (Model 1) and one for vigorous physical activity (Model 2). The results of the first model indicated that five predictors explained 47% of the variance in moderate physical activity time ( *R* ^2^_adjusted_=0.46, *F*(9,2863)=283.1, p<0.001). Older students ( *β*_age_=-0.73, p<0.001), as well as those who accumulated more moderate physical activity ( *β*=-0.71, p<0.001) and sitting time prior to confinement ( *β*=-0.01, p<0.001), had significantly less reduction in their moderate physical activity levels during confinement. It was found that active transportation time predicted a larger reduction in moderate physical activity ( *β*=0.16, p<0.001), as did vigorous physical activity prior to confinement ( *β*=0.07, p<0.001). These results mean that the more time spent in active transport and vigorous physical activity before confinement, the greater the reduction in moderate physical activity levels during confinement.

**Table 2.** Multiple regression analysis of changes in physical activity levels during confinement.

Variables	Change of MPA (Model 1)	95% CI		p-value	Change of VPA (Model 2)	95% CI		p-value
	β (SE)	LL	UL		β (SE)	LL	UL	
Intercept	35.26(6.13)	23.23	47.29	1.01e-08 ***	34.07(7.00)	20.24	47.91	1.44e-06 ***
Sex/Women	-1.63(1.00)	-0.32	3.60	0.102	-1.64(1.15)	-3.90	0.61	0.153
Age	-0.73(0.27)	-1.28	-018.	0.008 **	-0.58(0.32)	-1.21	0.04	0.069
MPA min/day (prior confinement)	-0.71(0.01)	-0.74	-0.69	<2e-16 ***	0.05(0.01)	0.01	0.08	0.002 **
VPA min/day (prior confinement)	0.07(0.01)	0.05	0.09	3.63e-10 ***	-0.95(0.01)	-0.98	-0.93	<2e-16 ***
Active transportation min/trip (without confinement)	0.16(0.02)	0.10	0.21	5.55e-09 ***	0.03(0.03)	-0.02	0.09	0.234
Sitting min/day (prior confinement)	-0.01(0.003)	-0.02	-0.008	1.61e-06 ***	-0.001(0.003)	-0.008	0.005	0.614
Uninterrupted sitting min/day (without confinement)	-0.005(0.007)	-0.02	-0.009	0.471	-0.002(0.009)	-0.01	0.01	0.820
Screen min/day (prior confinement)	0.002(0.003)	-0.003	0.008	0.374	-0.008(0.003)	-0.01	-0.001	0.018 *
Screen studying min/day (without confinement)	0.001(0.003)	-0.005	0.008	0.708	0.002(0.004)	-0.005	0.01	0.563
Observations	2873				2873			
R ^2^/adj R ^2^	.470/.469				.645/.644			
F-statistics	283.1			< 2.2e-16	580			<2.2e-16
AIC	26960.02				27762.23			

MPA=Moderate physical activity; VPA=Vigorous physical activity; LL=Lower Limit; UL=Upper Limit; AIC=Akaike information criterion. Significance codes:

* p<0.05;

** p<0.01;

*** p<0.001.

The second model showed that three predictors explained 64% of the variance in vigorous physical activity ( *R* ^2^_adjusted_=0.64, *F*(9,2863)=580, p<0.001). Students who engaged in vigorous physical activity ( *β*=-0.95, p<0.001) for more minutes prior to confinement showed significantly less reduction in vigorous physical activity during confinement. Conversely, students who presented higher amounts of moderate physical activity prior to confinement ( *β*=0.05, p<0.01) showed a significantly greater reduction in their levels of vigorous physical activity.

*H2*: The second hypothesis was that the greater the uninterrupted sitting time prior to confinement in students, the greater the increase in the amount of time they would spend sitting during confinement.

In the case of our second hypothesis, a multiple linear regression ( Table 3) showed four predictors (Model 3) explaining 49% of the variance in students’ sitting time ( *R* ^2^_adjusted_=0.49, *F*(9,2863)=318.8, p<0.001). Students’ siting time ( *β*=-0.97, p<0.001) and screen time studying ( *β*=-0.07, p=0.001) prior to confinement significantly predicted a smaller increase in sitting time during confinement. Conversely, the greater the uninterrupted sitting ( *β*=0.62, p<0.001) and screen time ( *β*=0.29, p<0.001) prior to confinement, the greater the increases in sitting time during confinement.

**Table 3.** Multiple regression models for changes in sitting and screen time during confinement.

Variables	Change of sitting time (Model 3)	p-value	95% CI		Change of screen time (Model 4)	95% CI		p-value
	β (SE)		LL	UL	β (SE)	LL	UL	
Intercept	501.11(40.45)	<2e-16 ^***^	421.78	580.43	405.80(37.34)	332.57	479.03	<2e-16 ^***^
Sex/Men	1.14(6.60)	0.862	-11.81	14.09	2.36(6.09)	-9.59	14.32	0.698
Age	-0.18(1.84)	0.921	-3.79	3.43	-0.68(1.70)	-4.02	2.64	0.685
MPA min/day (prior to confinement)	0.02(0.09)	0.791	-0.16	0.21	0.06(0.08)	-0.11	0.23	0.490
VPA min/day (prior to confinement)	-0.006(0.07)	0.931	-0.15	0.14	-0.07(0.07)	-0.21	0.06	0.266
Active transportation min/trip (without confinement)	-0.31(0.18)	0.083	-0.66	0.04	-0.52(0.16)	-0.85	-0.19	0.001 ^**^
Sitting min/day (prior to confinement)	-0.97(0.01)	<2e-16 ^***^	-1.01	-0.93	0.01(0.01)	-0.02	0.04	0.585
Uninterrupted sitting min/day (without confinement)	0.62(0.05)	<2e-16 ^***^	0.52	0.72	0.43 (0.04)	0.33	0.52	<2e-16 ^***^
Screen min/day (prior to confinement)	0.29(0.02)	<2e-16 ^***^	0.25	0.33	-0.45(0.01)	-0.48	-0.41	<2e-16 ^***^
Screen studying min/day (prior to confinement)	-0.07(0.02)	0.001 ^**^	-0.122	-0.02	-0.09(0.02)	-0.14	-0.05	7.65e-06 ^***^
Observations	2873				2873			
R ^2^/adj R ^2^	.500/.499				.200/.197			
F-statistics	318.8	<2.2e-16			79.54			<2.2e-16
AIC	37796.33				37337.03			

MPA=Moderate physical activity; VPA=Vigorous physical activity; LL=Lower Limit; UL=Upper Limit; AIC=Akaike information criterion. Significance codes:

* p<0.05;

** p<0.01;

*** p<0.001.

*H3*: The third hypothesis was for screen time; due to the increase in online courses during confinement, the longer the screen time studying prior to confinement in students, the greater the increase in their screen time during confinement.

Regressions ( Table 3) showed that four predictors explained 20% of the variance in screen time during confinement ( *R* ^2^_adjusted_=0.19, *F*(9,2863)=79.54, p<0.001). Active transportation ( *β*=-0.52, p<0.01), screen time prior to confinement ( *β*=-0.45, p<0.001), and screen time studying ( *β*=-0.09, p<0.001) predicted a lower increase in screen time during confinement. Only uninterrupted sitting ( *β*=0.43, p<0.001) significantly predicted a higher increase of screen time during confinement (Model 4).

*H4*: Finally, we hypothesised that there would be no relationship between active commuting, sitting time and screen time during confinement.

Multiple regression results on changes in moderate physical activity showed that the reduction in moderate physical activity levels was less significant for students who spent more time sitting prior to confinement ( *β*=-0.01, p<0.001). Model 2 showed no relationship between vigorous physical activity and either sitting or screen time. Model 3 showed no relationship between physical activity behaviour and sitting time or screen time. Finally, there was a negative relationship between the increase of screen time during confinement and active transportation minutes prior to confinement (Model 4). Students who adopted active transportation showed a less significant increase in screen time during confinement ( *β*=-0.52, p=0.001).

## Discussion

The main objective of this study was to determine whether the adoption of active transportation, uninterrupted sitting time, and screen study time predicted changes in physical activity, sitting time and screen time in students during confinement.

Studies on confinement and its effects on university students showed that it reduced physical activity levels in this population with at least two elements: the levels of physical activity before confinement and the age of the students. In fact, the reductions in physical activity levels were more significant for the more active students prior to confinement. ^43^ Moreover, these students are also the ones who used active transport to get to university before confinement. Thus, our results confirm that adopting active transport contributes to university students’ physical activity levels. ^7^ Students who adopted active transportation were more active before confinement than those who did not, which is consistent with the literature. ^2^ In addition, among those students who adopt active transport, those who have longer commuting times are also those who further decrease their physical activity levels. It is therefore necessary not only to look at total physical activity and active transport times, but also to look in detail at journey times. ^10^ Another detail of active transport could be whether students walk, cycle or combine these modes of transport. The study by Yang and colleagues ^10^ showed that men tend to cycle and women tend to walk.

In this study, age was a determining factor in reducing physical activity levels during confinement. The youngest students reduced their physical activity levels the most, which is consistent with the literature. ^5^ Thus, the year of training could have an influence; as the years of training progress, students decrease their physical activity levels. Therefore, future studies on physical activity levels in university students should consider the year of training.

For this reason, considering age and physical activity levels before confinement, in our study, an amplifying effect of active transportation time on reducing students’ physical activity levels during confinement was hypothesised and confirmed. Additionally, this study showed that the accumulation patterns of active transport allow us to identify individual differences and better adapt the recommendations to promote physical activity in university students in case of new confinements.

Students who spent time undertaking active transportation to university and vigorous physical activity levels prior to confinement predicted a significant reduction in moderate physical activity levels during confinement. However, active transportation time does not predict changes in vigorous physical activity levels during the confinement period. Active transportation is considered a physical activity characterised by light to moderate intensity ^44^; the students in this study adopted walking to the university prior to confinement.

Moreover, active transportation could be part of students’ daily routines, given that the city the study took place in has high active transportation rates (Walk Score ^®^ city of Villeurbanne: 89 errands can be accomplished on foot and very bikeable: 70-89). ^45^ However, the suspension of campus lectures reduced active commuting, decreasing overall moderate physical activity levels. This factor explains why active transportation time did not predict a greater reduction in vigorous physical activity levels. In addition, students were inactive and spent no more than 30 minutes per day in physical activity, coupled with reduced movement perimeter during confinement ( *i.e.*, 5 km around the house).

It might have been expected that levels of moderate and vigorous physical activity before confinement would explain the changes in the respective levels during confinement. However, the results only showed that vigorous physical activity levels before confinement had a smaller reduction in time spent in vigorous activities during confinement. With regard to moderate physical activity levels, the reduction of that time during confinement also reduced vigorous activity levels. This result could be due to the fact that in order to reach vigorous intensity, it is necessary to go through moderate activity levels. ^46^ This is of concern because, for students who were more inclined to engage in vigorous physical activity prior to confinement, their practice during confinement was significantly reduced. This reduction could lead to negative health outcomes, a serious concern that warrants further attention.

Our second hypothesis was confirmed; the longer the uninterrupted time spent in a seated position before confinement, the more the students increased their time in that position during confinement. Furthermore, this increased time was exacerbated by the screen’s use during confinement. This factor, combined with sitting time, indicated that the students who spent the most time sitting without interruption were those who spent the most time in front of screens. ^18^ This phenomenon increased during confinement in general population and as our study showed, also in students. ^34^ Uninterrupted sitting time has been identified as a health risk factor in adults. ^16^ This behaviour pattern is particularly marked in university students who accumulate this time during their lectures. ^47^ Consequently, the point is the accumulation pattern of sitting time ( *i.e.*, how this time accumulates). For example, two students might accumulate six hours in total, but one will do so in three periods of two hours each and another in six periods of one hour.

Regarding the positive association between sitting time and screen use, it is likely that the online courses during the pandemic period have increased the association between screen use and sitting in university students, increasing the use of screens to study. ^21^ Similarly, screen use in students could be interpreted as a proxy for time spent sitting.

The third hypothesis postulated that the more time students used screens prior to confinement, the more they would increase this time during confinement. The results did not confirm this hypothesis; on the contrary, the more time spent using screens prior to confinement, the smaller the increase in this behaviour. One possible explanation could be that high time spent using screens did not undergo significant modifications since screen-based devices have become pervasive in students’ lives. ^21^ Likewise, the time students spent using screens could be independent of the confinement factor. However, screen time would not be independent of the uninterrupted time spent sitting since the longer the prolonged periods of this behaviour, the greater the increase in screen time. Thus, it could be postulated that students defined as prolongers of sitting time ^29^ would be more prone to prolonged use of screens and vice-versa.

The last hypothesis postulated that there would be no relationship between active transportation times, sitting, and screen time was partially confirmed. The relationship between moderate physical activity and students’ sitting time showed that low levels of physical activity could coexist with high levels of sitting time in students. This inactive-sedentary profile should be the most alarming health risk factor. Inactivity itself results in low levels of physical and mental health such as such as stress, anxiety, depression, ^14^ but if it is associated with high levels of sitting time, there is a potentially higher risk of suffering from metabolic diseases or exacerbating risk for mortality. ^28^ Another element highlighted is the “protective” effect of time spent in active transportation. This study highlight that the more time students adopted active transportation, the less they increased screen use in confinement. This phenomenon could reflect a negative association between the two behaviours and that somehow, screen time would be displaced by active transportation time. ^30^ Similarly, students who adopt active transportation are generally more active people ^7^ and spend less time using screens.

## Limitations

The present study is not free of limitations. Behaviours were measured via questionnaires, leading to the overestimation of physical activity and underestimation of sitting time. This phenomenon is associated with social desirability. There could be a bias of recall about before the confinement. The students who could answer this questionnaire had access to the internet and were enrolled in the health seminars offered during the pandemic. A potential selection bias may be present, as students who participated in the health webinar may represent a portion of the university population already more sensitive to the topic of physical activity. There were several measures to continue working at universities in France. Few universities organize face-to-face teaching. Thus, this study only represents part of the student population of French universities. Regional differences in lockdown policies may have influenced the results, necessitating caution when interpreting the data at a national level.

Future studies on physical activity levels, the adoption of active transport and screen use in university students could include socio-economic and cultural factors.

## Conclusions

Our study confirms that active transportation is the primary source of physical activity in the daily lives of university students. Thus, active transport at university should be a health priority, including its promotion on university campuses. Moreover, access to cycle paths and footpaths near universities would facilitate the adoption of this behaviour by students. Similarly, the time young people spend studying contributes to the accumulation of high levels of sitting time, which screens could exacerbate. In addition, screens’ ubiquity and association with sedentary behaviours could induce prolonged sitting positions, generating patterns of behaviour that are riskier for students’ health.

For the reasons outlined above, implementing short breaks from sitting time during university courses should be a policy to apply at universities. Similarly, online courses in which screens are the main element should include recommendations for teachers and students to interrupt the time students spend in front of these devices in sedentary positions. Therefore, the study of online courses and e-learning on physical activity levels and sitting time must be determined.

## Data availability

### Underlying data

Figshare: Uni_dataCovid19 (spreadsheet data of questionnaire results). https://doi.org/10.6084/m9.figshare.19583821. ^42^

### Extended data

Figshare: Questionnaire Uni (study questionnaire), https://doi.org/10.6084/m9.figshare.19778761.v1. ^37^

Figshare: GAM_RGraphs_uni (GAM graphs to examine subgroups and interactions). https://doi.org/10.6084/m9. figshare.19630434. ^40^

Data are available under the terms of the Creative Commons Attribution 4.0 International license (CC-BY 4.0).

## References

[1] Lopez-Valenciano A Suarez-Iglesias D Sanchez-Lastra MA : Impact of COVID-19 Pandemic on University Students’ Physical Activity Levels: An Early Systematic Review. *Front. Psychol.* 2020;11:624567. 33519653 10.3389/fpsyg.2020.624567 PMC7845570

[2] Genin PM Lambert C Larras B : How Did the COVID-19 Confinement Period Affect Our Physical Activity Level and Sedentary Behaviors? Methodology and First Results From the French National ONAPS Survey. *J. Phys. Act. Health.* 2021;18(3):296–303. 10.1123/jpah.2020-0449 33581686

[3] Romero-Blanco C Rodriguez-Almagro J Onieva-Zafra MD : Physical Activity and Sedentary Lifestyle in University Students: Changes during Confinement Due to the COVID-19 Pandemic. *Int. J. Environ. Res. Public Health.* 2020;17(18). 10.3390/ijerph17186567 32916972 PMC7558021

[4] Motevalli M Drenowatz C Wirnitzer KC : Changes in physical activity during the COVID-19 lockdown based on the sociodemographic profile of 5569 students and academic staff of Austrian universities. *Public Health.* 2023;219:102–109. 10.1016/j.puhe.2023.04.003 37149990 PMC10073584

[5] Meyer J Herring M McDowell C : Joint prevalence of physical activity and sitting time during COVID-19 among US adults in April 2020. *Prev. Med. Rep.* 2020; (20):101256. Epub 2020 Nov 27. Erratum in: Prev Med Rep. 202106; 22: 101354, 20. 10.1016/j.pmedr.2020.101256 33282638 PMC7695441

[6] Flint E Webb E Cummins S : Change in commute mode and body-mass index: prospective, longitudinal evidence from UK Biobank. *Lancet Public Health.* 2016;1(2):e46–e55. 10.1016/S2468-2667(16)30006-8 28299370 PMC5341146

[7] Crist K Brondeel R Tuz-Zahra F : Correlates of active commuting, transport physical activity, and light rail use in a university setting. *J. Transp. Health.* 2021;20:100978. 10.1016/j.jth.2020.100978

[8] Sisson SB Tudor-Locke C : Comparison of cyclists’ and motorists’ utilitarian physical activity at an urban university. *Prev. Med.* 2008;46(1):77–79. 10.1016/j.ypmed.2007.07.004 17707076

[9] Sahlqvist S Song Y Ogilvie D : Is active travel associated with greater physical activity? The contribution of commuting and non-commuting active travel to total physical activity in adults. *Prev. Med.* 2012;55(3):206–211. 10.1016/j.ypmed.2012.06.028 22796629 PMC3824070

[10] Yang L Panter J Griffin SJ : Associations between active commuting and physical activity in working adults: cross-sectional results from the Commuting and Health in Cambridge study. *Prev. Med.* 2012;55(5):453–457. 10.1016/j.ypmed.2012.08.019 22964003 PMC3863955

[11] Hanson S Jones A : Is there evidence that walking groups have health benefits? A systematic review and meta-analysis. Br. J. Sports Med. 2015 Jun;49(11):710–715. 10.1136/bjsports-2014-094157 25601182 PMC4453623

[12] Colberg SR Sigal RJ Fernhall B : American College of Sports Medicine; American Diabetes Association. Exercise and type 2 diabetes: the American College of Sports Medicine and the American Diabetes Association: joint position statement. *Diabetes Care.* 2010 Dec;33(12):e147–e167. 10.2337/dc10-9990 21115758 PMC2992225

[13] Zhao W Ukawa S Kawamura T : Health Benefits of Daily Walking on Mortality Among Younger-Elderly Men With or Without Major Critical Diseases in the New Integrated Suburban Seniority Investigation Project: A Prospective Cohort Study. J. Epidemiol. 2015;25(10):609–616. 10.2188/jea.JE20140190 26155815 PMC4626390

[14] Edwards MK Loprinzi PD : Experimental effects of brief, single bouts of walking and meditation on mood profile in young adults. *Health Promot. Perspect.* 2018 Jul 7;8(3):171–178. 10.15171/hpp.2018.23 30087839 PMC6064756

[15] Bull FC Al-Ansari SS Biddle S : World Health Organization 2020 guidelines on physical activity and sedentary behaviour. *Br. J. Sports Med.* 2020;54(24):1451–1462. 10.1136/bjsports-2020-102955 33239350 PMC7719906

[16] Bellettiere J Winkler EAH Chastin SFM : Associations of sitting accumulation patterns with cardio-metabolic risk biomarkers in Australian adults. *PLoS One.* 2017;12(6):e0180119. 10.1371/journal.pone.0180119 28662164 PMC5491133

[17] Patterson R McNamara E Tainio M : Sedentary behaviour and risk of all-cause, cardiovascular and cancer mortality, and incident type 2 diabetes: a systematic review and dose response meta-analysis. *Eur. J. Epidemiol.* 2018;33(9):811–829. 10.1007/s10654-018-0380-1 29589226 PMC6133005

[18] Moulin MS Irwin JD : An assessment of sedentary time among undergraduate students at a Canadian university. *Int. J. Exerc. Sci.* 2017;10(8):1116–1129.

[19] Diaz KM Howard VJ Hutto B : Patterns of Sedentary Behavior and Mortality in U.S. Middle-Aged and Older Adults: A National Cohort Study. *Ann. Intern. Med.* 2017;167(7):465–475. 10.7326/M17-0212 28892811 PMC5961729

[20] Montagni I Guichard E Carpenet C : Screen time exposure and reporting of headaches in young adults: A cross-sectional study. *Cephalalgia.* 2016;36(11):1020–1027. 10.1177/0333102415620286 26634831

[21] Castro O Bennie J Vergeer I : How sedentary are university students? A systematic review and meta-analysis. *Prev. Sci.* 2020;21(3):332–343. 10.1007/s11121-020-01093-8 31975312

[22] Castro O Vergeer I Bennie J : Using the behavior change wheel to understand why university students’ prolonged sitting time and identify potential intervention strategies. *Int. J. Beh. Med.* 2021;28(3):360–371. 10.1007/s12529-020-09926-0 32820465

[23] Clark BK Healy GN Winkler EA : Relationship of television time with accelerometer-derived sedentary time: NHANES. *Med. Sci. Sports Exerc.* 2011;43(5):822–828. 10.1249/MSS.0b013e3182019510 20980928 PMC8477752

[24] Hamer M Yates T Demakakos P : Television viewing and risk of mortality: exploring the biological plausibility. *Atherosclerosis.* 2017;263:151–155. 10.1016/j.atherosclerosis.2017.06.024 28645071

[25] Rhodes RE Mark RS Temmel CP : Adult sedentary behavior: a systematic review. *Am. J. Prev. Med.* 2012;42(3):e3–e28. 10.1016/j.amepre.2011.10.020 22341176

[26] Ekelund U Steene-Johannessen J Brown WJ : Does physical activity attenuate, or even eliminate, the detrimental association of sitting time with mortality? A harmonised meta-analysis of data from more than 1 million men and women. *Lancet.* 2016;388(10051):1302–1310. 10.1016/S0140-6736(16)30370-1 27475271

[27] Castro O Bennie J Vergeer I : Correlates of sedentary behaviour in university students: A systematic review. *Prev. Med.* 2018;116:194–202. 10.1016/j.ypmed.2018.09.016 30266213

[28] Dunstan DW Dogra S Carter SE : Sit less and move more for cardiovascular health: emerging insights and opportunities. *Nat. Rev. Cardiol.* 2021;18(9):637–648. 10.1038/s41569-021-00547-y 34017139

[29] Saunders TJ McIsaac T Douillette K : Sedentary behaviour and health in adults: an overview of systematic reviews. *Appl. Physiol. Nutr. Metab.* 2020;45(10 (Suppl. 2)):S197–S217. 10.1139/apnm-2020-0272 33054341

[30] Stamatakis E Gale J Bauman A : Sitting Time, Physical Activity, and Risk of Mortality in Adults. *J. Am. Coll. Cardiol.* 2019;73(16):2062–2072. 10.1016/j.jacc.2019.02.031 31023430

[31] Heller S Kalo K Werner AM : Sedentary time of university students before and during the COVID-19 pandemic: Risk groups and pre-pandemic predictors using cross-sectional and longitudinal data. *Front. Public Health.* 2023;11:1138442. 10.3389/fpubh.2023.1138442 37089509 PMC10113436

[32] Trott M Driscoll R Irlado E : Changes and correlates of screen time in adults and children during the COVID-19 pandemic: A systematic review and meta-analysis. *EclinicalMedicine.* 2022;48: 101452. 10.1016/j.eclinm.2022.101452 35615691 PMC9122783

[33] López-Valenciano A Suárez-Iglesias D Sanchez-Lastra MA : Impact of COVID-19 Pandemic on University Students’ Physical Activity Levels: An Early Systematic Review. *Front. Psychol.* 2021;11: 624567. 10.3389/fpsyg.2020.624567 33519653 PMC7845570

[34] Guo YF Liao MQ Cai WL : Physical activity, screen exposure and sleep among students during the pandemic of COVID-19. *Sci. Rep.* 2021 Apr 20;11(1):8529. 10.1038/s41598-021-88071-4 33879822 PMC8058040

[35] Rillamas-Sun E LaMonte MJ Evenson KR : The Influence of Physical Activity and Sedentary Behavior on Living to Age 85 Years Without Disease and Disability in Older Women. J. Gerontol. A Biol. Sci. Med. Sci. 2018Oct 8;73(11):1525–1531. 10.1093/gerona/glx222 29165626 PMC6175029

[36] Silva GO Cunha PM Oliveira MD : Patterns of sedentary behavior in adults: A cross-sectional study. *Front. Cardiovasc. Med.* 2023Mar;10:1116499. 10.3389/fcvm.2023.1116499 36993993 PMC10042287

[37] Marchant G : Questionnaire Uni. figshare. *Figure. [Dataset].* 2022. 10.6084/m9.figshare.19778761.v1

[38] Craig CL Marshall AL Sjostrom M : International physical activity questionnaire: 12-country reliability and validity. *Med. Sci. Sports Exerc.* 2003;35(8):1381–1395. 10.1249/01.MSS.0000078924.61453.FB 12900694

[39] Wood SN : *Generalized Additive Models: An Introduction with R.* (2nd ed.). Chapman and Hall/CRC;2017. 10.1201/9781315370279

[40] Marchant G : GAM_RGraphs_uni. figshare. *Figure. [Dataset].* 2022. 10.6084/m9.figshare.19630434

[41] Bates D Mächler M Bolker B : Fitting Linear Mixed-Effects Models Using lme4. *J. Stat. Softw.* 2015;67(1):1–48. 10.18637/jss.v067.i01

[42] Marchant G : Uni_dataCovid19. figshare. *[Dataset].* 2022. 10.6084/m9.figshare.19583821

[43] McCarthy H Potts HWW Fisher A : Physical Activity Behavior Before, During, and After COVID-19 Restrictions: Longitudinal Smartphone-Tracking Study of Adults in the United Kingdom. *J. Med. Internet Res.* 2021 Feb 3;23(2): e23701. 10.2196/23701 33347421 PMC7861037

[44] Ainsworth BE Haskell WL Herrmann SD : 2011 Compendium of Physical Activities: a second update of codes and MET values. *Med. Sci. Sports Exerc.* 2011;43(8):1575–1581. 10.1249/MSS.0b013e31821ece12 21681120

[45] Hall CM Ram Y : Walk score ^®^ and its potential contribution to the study of active transport and walkability: A critical and systematic review. *Transp. Res. Part D: Transp. Environ.* 2018;61:310–324. 10.1016/j.trd.2017.12.018

[46] Eijsvogels TM George KP Thompson PD : Cardiovascular benefits and risks across the physical activity continuum. *Curr. Opin. Cardiol.* 2016;31(5):566–571. 10.1097/HCO.0000000000000321 27455432

[47] Deliens T Deforche B De Bourdeaudhuij I : Determinants of physical activity and sedentary behaviour in university students: a qualitative study using focus group discussions. *BMC Public Health.* 2015;15:201. 10.1186/s12889-015-1553-4 25881120 PMC4349731

