# Supplementary material for: Behavioural patterns of university students during the COVID-19 pandemic: A cross-sectional study of the effects of active transportation, uninterrupted sitting time, and screen use on physical activity and sitting time
Source: F1000Res. 2025 Jan 29;11:568. Originally published 2022 May 25. [Version 3] doi: 10.12688/f1000research.117843.3 (PMC11615430; doi:10.12688/f1000research.117843.3)
Supplement: Peer review history

# Reviewer report: Reviewer response for version 2

**DOI:** 10.5256/f1000research.158961.r322483

**Published:** 2024-12-03

**Authors:** Pierluigi Diotaiuti

**Affiliations:** 1 Department of Human Sciences, Society and Health, University of Cassino and Southern Lazio, Cassino, Italy

I sincerely thank you for giving me the opportunity to review your study titled *"Behavioural patterns of university students during the COVID-19 pandemic: A cross-sectional study of the effects of active transportation, uninterrupted sitting time, and screen use on physical activity and sitting time."* This manuscript addresses a highly relevant topic, rigorously exploring the behavioral changes of university students during the pandemic lockdown. The methodological approach, combined with the large sample size and detailed statistical analysis, makes this work a valuable contribution to understanding the pandemic's impact on students' physical and sedentary behaviors.

 Strengths of the Study

1. **Originality of the Topic**: The study focuses on a current and under-explored subject in scientific literature: the pandemic's effect on behaviors related to active transportation, uninterrupted sitting time, and screen use among university students.
2. **Strong Methodological Structure**: The use of the IPAQ questionnaire and multiple regression models ensures robust data collection. The decision to examine various variables, such as active transportation and screen time for studying, provides a comprehensive and well-rounded analysis.
3. **Clarity of Presentation**: The text is generally clear and well-structured, with appropriate scientific terminology and a thorough description of methods and results.

However, during the review process, I identified several areas where improvements could further enhance the value of the manuscript. Below, I provide detailed suggestions, specifying the exact lines and the modifications to be made.

 Suggested Modifications

1. **Abstract**
  - **Lines 19-22**: The sentence *"The closure of universities due to the COVID-19 pandemic may alter the behaviour of students"* can be made more precise. I suggest replacing it with: *"The closure of universities due to the COVID-19 pandemic significantly affected students' behaviors, particularly regarding physical activity, sitting time, and screen use."* This wording clarifies the direct impact described in the study.
  - **Lines 23-24**: As previously mentioned, replace *"lockdown"* with *"confinement"* to maintain terminological consistency.
  - **Lines 26-27**: It is mentioned that the sample completed the International Physical Activity Questionnaire (IPAQ), but some key details are missing. Please add the average completion time and specify how informed consent was obtained. I suggest: *"Participants completed the IPAQ in an average time of around 15 minutes, after providing digital informed consent."*
2. **Introduction**
  - **Lines 62-65**: The sentence *"The decrease in physical activity levels was more significant for people who were more active before COVID-19, men and younger adults (ages 18-34) compared to older adults."* needs more clarity and support. You might expand by explaining why these categories were more affected, such as referencing specific behavioral or socio-economic factors.
  - **Lines 71-73**: It would be helpful to expand the section on active transportation, including studies that support the importance of this form of movement for mental and physical health during confinement.
3. **Methods**
  - **Lines 105-106**: The description of the email invitation to students could be improved by adding more detail. I suggest clarifying if the invitation was sent multiple times, whether incentives were used to encourage participation, and the final response rate. Add: *"The survey invitation was sent twice to increase response rates, and participants were offered access to the final research results as a non-monetary incentive."*
  - **Lines 116-117**: It would be useful to specify how missing data were handled. I suggest: *"Missing data were handled by excluding incomplete cases from the dataset, avoiding the use of multiple imputations."*
4. **Results**
  - **Lines 146-150**: In this section, where the regression model results are reported, it would be helpful to provide a more detailed interpretation of the β coefficients. For example, regarding active transportation, explain more clearly how transport time predicted a greater reduction in moderate physical activity, specifying the practical implications of these findings.
  - **Lines 160-162**: The description of the sample in terms of IPAQ could be improved by adding a reflection on the importance of including socio-economic or cultural categories in the analysis models.
5. **Discussion**
  - **Lines 192-195**: The paragraph discussing the importance of active transportation could benefit from more emphasis on the mental health benefits in addition to physical ones. I suggest including a reference to the literature that highlights how physical activity positively affects psychological well-being, especially in a confinement context.
  - **Lines 203-207**: It would be helpful to clarify the interpretation of the results related to vigorous versus moderate physical activity. The explanation provided seems too concise and may confuse the reader. I suggest expanding, specifying how the intensity of physical activity influenced behaviors during confinement.

In the **limitations** section, although some crucial points have been identified, there are other aspects that deserve further discussion:

1. **Lines 263-265**: While social desirability bias is mentioned, a more in-depth reflection on selection bias is needed. Those who participated in the health webinar might have already been more sensitive to the topic of physical activity compared to the general student population. I suggest adding: *"A potential selection bias may be present, as students who participated in the health webinar may represent a portion of the university population already more sensitive to the topic of physical activity."*
2. **Lines 267-269**: You might consider the potential influence of regional differences within France, where some areas may have experienced stricter restrictions than others. Add a comment: *"Regional differences in lockdown policies may have influenced the results, necessitating caution when interpreting the data at a national level."*
3. **Section "Screen time and sedentary behaviour" (Lines 143-146)**: This section is somewhat repetitive. Some concepts, such as the correlation between screen time and sedentariness, have already been mentioned in other parts. I suggest condensing and merging these sections to avoid redundancy.

In reviewing your manuscript, I suggest including the following reference:

 Diotaiuti P, et al., 2021 (Ref 1)

 This article could be particularly relevant to enrich the discussion on the validation of psychometric tools used to measure behaviors and psychological variables related to physical activity and sedentary behavior. I recommend inserting this citation in the following sections of your manuscript:

1. **Methods Section**, specifically where you describe the validation and reliability of the tools used to measure sedentary behaviors and screen time. You could add a sentence such as: *"To ensure the accuracy and validity of behavioral measurements, we considered the validation of similar tools in other contexts, as demonstrated in the study by Diotaiuti et al. (2021), which confirmed the psychometric robustness of temporal focus measurement scales."*
2. **Discussion Section**, when addressing the importance of using validated and reliable tools in research on behaviors during confinement. You might add: *"The importance of using validated tools to measure psychological factors is well documented in the literature, as illustrated by the study of Diotaiuti et al. (2021) [Ref 1], which demonstrated the psychometric validity of the Temporal Focus Scale in a university setting."*

Is the work clearly and accurately presented and does it cite the current literature?

Yes

If applicable, is the statistical analysis and its interpretation appropriate?

Yes

Are all the source data underlying the results available to ensure full reproducibility?

Yes

Is the study design appropriate and is the work technically sound?

Yes

Are the conclusions drawn adequately supported by the results?

Partly

Are sufficient details of methods and analysis provided to allow replication by others?

Yes

Reviewer Expertise:

In the context of reviewing and providing feedback on research articles, my areas of research focus on physical activity, sedentary behavior, public health interventions, and behavioral patterns during pandemics. This includes understanding how environmental factors such as confinement and lifestyle changes (e.g., screen use, sitting time, active transportation) affect physical and mental health outcomes, particularly among vulnerable populations like students. Additionally, I am engaged in methodological assessments related to cross-sectional and longitudinal studies, emphasizing data collection tools, psychometric validations, and regression models.

I confirm that I have read this submission and believe that I have an appropriate level of expertise to confirm that it is of an acceptable scientific standard, however I have significant reservations, as outlined above.

## References

[1] : Validation study of the Italian version of Temporal Focus Scale: psychometric properties and convergent validity. *BMC Psychology* .2021;9(1) : 10.1186/s40359-020-00510-5 10.1186/s40359-020-00510-5 33522963 PMC7851924

# Response

**Published:** 2025-01-22

**Authors:** Gonzalo Marchant

**Affiliations:** University of Rouen Normandy, France

Suggested Modifications

1. **Abstract**
  - **Lines 19-22**: The sentence *"The closure of universities due to the COVID-19 pandemic may alter the behaviour of students"* can be made more precise. I suggest replacing it with: *"The closure of universities due to the COVID-19 pandemic significantly affected students' behaviors, particularly regarding physical activity, sitting time, and screen use."* This wording clarifies the direct impact described in the study.

**Answer**: We replaced this phrase.

-
  - **Lines 23-24**: As previously mentioned, replace *"lockdown"* with *"confinement"* to maintain terminological consistency.

**Answer: **We replaced lockdown with confinement.

-
  - **Lines 26-27**: It is mentioned that the sample completed the International Physical Activity Questionnaire (IPAQ), but some key details are missing. Please add the average completion time and specify how informed consent was obtained. I suggest: *"Participants completed the IPAQ in an average time of around 15 minutes, after providing digital informed consent."*

**Answer: **We have included your suggestion.

  

1. **Introduction**
  - **Lines 62-65**: The sentence *"The decrease in physical activity levels was more significant for people who were more active before COVID-19, men and younger adults (ages 18-34) compared to older adults."* needs more clarity and support. You might expand by explaining why these categories were more affected, such as referencing specific behavioral or socio-economic factors.

**Answer**: The cited study (5) shows that the determining factor in this decline was age. To make it clear that the age category was the key factor in this decline, we have added age >34 years. The scientific literature shows that physical activity levels tend to decrease with advancing age.

-
  - **Lines 71-73**: It would be helpful to expand the section on active transportation, including studies that support the importance of this form of movement for mental and physical health during confinement.

**Answer**: We have included active transport adoption's mental and physical effects. See page 4, lines 14-18, and references 11 to 14.

1. **Methods**
  - **Lines 105-106**: The description of the email invitation to students could be improved by adding more detail. I suggest clarifying if the invitation was sent multiple times, whether incentives were used to encourage participation, and the final response rate. Add: *"The survey invitation was sent twice to increase response rates, and participants were offered access to the final research results as a non-monetary incentive."*

**Answer: **We added your suggestion in the method section.

-
  - **Lines 116-117**: It would be useful to specify how missing data were handled. I suggest: *"Missing data were handled by excluding incomplete cases from the dataset, avoiding the use of multiple imputations."*

**Answer: **We added your suggestion in the method section.

1. **Results**
  - **Lines 146-150**: In this section, where the regression model results are reported, it would be helpful to provide a more detailed interpretation of the β coefficients. For example, regarding active transportation, explain more clearly how transport time predicted a greater reduction in moderate physical activity, specifying the practical implications of these findings.

**Answer: **We explain the results of active transport and vigorous activity. Please see the results section Hypothesis 1. Page 9, lines 197-199.

-
  - **Lines 160-162**: The description of the sample in terms of IPAQ could be improved by adding a reflection on the importance of including socio-economic or cultural categories in the analysis models.

**Answer: **We did not include these factors in our questionnaire. We include this suggestion in our study's limitations, page 15, lines 349-351.

1. **Discussion**
  - **Lines 192-195**: The paragraph discussing the importance of active transportation could benefit from more emphasis on the mental health benefits in addition to physical ones. I suggest including a reference to the literature that highlights how physical activity positively affects psychological well-being, especially in a confinement context.

**Answer:** We included this reference, and we wrote a new phrase. See age 15, line 331.  

-
  - **Lines 203-207**: It would be helpful to clarify the interpretation of the results related to vigorous versus moderate physical activity. The explanation provided seems too concise and may confuse the reader. I suggest expanding, specifying how the intensity of physical activity influenced behaviors during confinement.

**Answer: **We rephrase this explanation. Please see page 14, Lines 290-299.

 In the **limitations** section, although some crucial points have been identified, there are other aspects that deserve further discussion:

1. **Lines 263-265**: While social desirability bias is mentioned, a more in-depth reflection on selection bias is needed. Those who participated in the health webinar might have already been more sensitive to the topic of physical activity compared to the general student population. I suggest adding: *"A potential selection bias may be present, as students who participated in the health webinar may represent a portion of the university population already more sensitive to the topic of physical activity."*

**Answer:** We have now included your suggestion.

1. **Lines 267-269**: You might consider the potential influence of regional differences within France, where some areas may have experienced stricter restrictions than others. Add a comment: *"Regional differences in lockdown policies may have influenced the results, necessitating caution when interpreting the data at a national level."*

**Answer:** We have now included your suggestion.

1. **Section "Screen time and sedentary behaviour" (Lines 143-146)**: This section is somewhat repetitive. Some concepts, such as the correlation between screen time and sedentariness, have already been mentioned in other parts. I suggest condensing and merging these sections to avoid redundancy.

**Answer:** We prefer to repeat this information as it is key to the importance of this relationship for university students, mainly because screens are increasingly being used in universities. Thanks for your suggestion.

 In reviewing your manuscript, I suggest including the following reference:

 Diotaiuti P, et al., 2021 (Ref 1)

 This article could be particularly relevant to enrich the discussion on the validation of psychometric tools used to measure behaviors and psychological variables related to physical activity and sedentary behavior. I recommend inserting this citation in the following sections of your manuscript:

1. **Methods Section**, specifically where you describe the validation and reliability of the tools used to measure sedentary behaviors and screen time. You could add a sentence such as: *"To ensure the accuracy and validity of behavioral measurements, we considered the validation of similar tools in other contexts, as demonstrated in the study by Diotaiuti et al. (2021), which confirmed the psychometric robustness of temporal focus measurement scales."*
2. **Discussion Section**, when addressing the importance of using validated and reliable tools in research on behaviors during confinement. You might add: *"The importance of using validated tools to measure psychological factors is well documented in the literature, as illustrated by the study of Diotaiuti et al. (2021) [Ref 1], which demonstrated the psychometric validity of the Temporal Focus Scale in a university setting."*

**Answer:** Thank you for your suggestion. However, I would like to point out that we used an already validated questionnaire (IPAQ) and secondly, we did not use psychological assessments. Moreover, I could not find the reference to which you refer.

# Reviewer report: Reviewer response for version 2

**DOI:** 10.5256/f1000research.158961.r246559

**Published:** 2024-02-22

**Authors:** Akhmad Habibi

**Affiliations:** 1 Universitas Jambi, Jambi, Indonesia

The authors have revised the work thoroughly, Congratulations.

 I support the indexing of the manuscript.

Is the work clearly and accurately presented and does it cite the current literature?

Yes

If applicable, is the statistical analysis and its interpretation appropriate?

Partly

Are all the source data underlying the results available to ensure full reproducibility?

Yes

Is the study design appropriate and is the work technically sound?

Partly

Are the conclusions drawn adequately supported by the results?

Partly

Are sufficient details of methods and analysis provided to allow replication by others?

Yes

Reviewer Expertise:

Statistics, educational technology

I confirm that I have read this submission and believe that I have an appropriate level of expertise to confirm that it is of an acceptable scientific standard.

# Reviewer report: Reviewer response for version 1

**DOI:** 10.5256/f1000research.129683.r180217

**Published:** 2023-07-20

**Authors:** Noémie Lienhart

**Affiliations:** 1 Sport Psychology, University of Nantes, Nantes, France

Overall, this study sought to determine whether the adoption of active transportation, uninterrupted sitting time, and screen study time predicted changes in physical activity, sitting time, and screen time in students during confinement. I think this has the potential to contribute to physical activity and sitting time fields. However, there are several concerns that I have with the manuscript. I have outlined them below, including general and specific comments.

General comments:

- The whole study is based on the closure of the universities and therefore the e-teaching during this second lockdown (October to December 2020). At that time, however, universities had the right to organize face-to-face teaching under some conditions. The situation was dependent on the faculty and even the year of study. If the authors do not have this information, they must consider this situation in their interpretation.
- Recall bias does not appear to be considered by the authors. However, prior to confinement data are collected during confinement and this bias can be very significant.
- Introduction: the interest and direction of hypothesis 4 were not discussed in the light of the literature in the introduction part.
- Methods: Were participants required to attend the webinar to receive the link? What was the content of the webinar? Depending on this, participants may have adapted their responses.
- Discussion: authors must make a real effort to present the practical applications of their work. Outside of the pandemic, what can we learn from this work?

Specific comments:

- Abstract: The wording of the objective is confusing. It is not the effect of the pandemic on pre-lockdown and lockdown that is being explored but the effect of active transportation, uninterrupted sitting time, and screen study time on physical activity and sitting time during the lockdown.
- Introduction: I don’t understand what hypothesis 3 is based on. The increase in online courses during confinement is a phenomenon independent of the screen time prior to confinement.
- Measures – Physical activity and Sitting time: Be careful of inconsistency. A sentence mentions that the data of the last seven days are requested while in the example it is mentioned “prior to confinement” and “during the confinement”.
- Measures: Some data were measured only on weekdays while others are measured in general. Why sitting time, uninterrupted sitting time, screen time, and screen time studying data were not measured separately for weekdays and weekends? We might hypothesize there are differences.
- Statistical analysis: Why sex and age were included in the regression model as control variables? You need to justify this in the introduction part.
- Statistical analyses: the 4th hypothesis is not mentioned.
- Results: “Students spent more than 10 hours per day using screens, of which approximately five hours were for …”. Please specify “during confinement” if this data refers to this period.
- Table 1: The data in the right-hand column is based on headcount, not frequency.
- Hypothesis: Please reformulate “Multiple regression models showed that when there was a negative effect in changes, it opposes the effect of confinement, if there is a positive change effect, it increases the impact of confinement.” for the lecturer.
- Tables 2 and 3: Does “habitual” corresponds to data during confinement? This term is not used in the manuscript.
- Results – H3: Is this the screen time to study **before confinement** that predicts a lower increase in screen time during confinement?

Is the work clearly and accurately presented and does it cite the current literature?

Partly

If applicable, is the statistical analysis and its interpretation appropriate?

Yes

Are all the source data underlying the results available to ensure full reproducibility?

Yes

Is the study design appropriate and is the work technically sound?

Partly

Are the conclusions drawn adequately supported by the results?

Yes

Are sufficient details of methods and analysis provided to allow replication by others?

Partly

Reviewer Expertise:

Sport psychology, elite athletes, parents, coaches, self determination theory, coping

I confirm that I have read this submission and believe that I have an appropriate level of expertise to confirm that it is of an acceptable scientific standard, however I have significant reservations, as outlined above.

# Response

**Published:** 2023-11-28

**Authors:** Gonzalo Marchant

**Affiliations:** University of Rouen Normandy, France

Dear Colleague,

 First of all, we appreciate your time and constructive comments so that we can improve our work.

 In the following lines, we detail each of the responses to your comments.

 Our response is presented after each of your comments.

General comments:

- The whole study is based on the closure of the universities and therefore the e-teaching during this second lockdown (October to December 2020). At that time, however, universities had the right to organize face-to-face teaching under some conditions. The situation was dependent on the faculty and even the year of study. If the authors do not have this information, they must consider this situation in their interpretation.

Answer 1: We are aware of this limit. However, we did not control it because the whole university was closed when the questionnaire was online. We include this factor as a limitation of our study.

  

- Recall bias does not appear to be considered by the authors. However, prior to confinement data are collected during confinement and this bias can be very significant.

Answer 2: We include this recall bias in the limits section.

  

- Introduction: the interest and direction of hypothesis 4 were not discussed in the light of the literature in the introduction part.

Answer 3: We complete this literature and include new arguments to make them more coherent with our fourth hypothesis. Please see the references Heller et al., 2023; Trott et al., 2022 (Fifth paragraph of introduction section).  

  

- Methods: Were participants required to attend the webinar to receive the link? What was the content of the webinar? Depending on this, participants may have adapted their responses.

Answer 4: The Webinar was about physical (in) activity and sedentary behaviours. The duration was 15 minutes. It was before questionnaires. Students received via email the link to the questionnaire after the Webinar. However, the response turnaround time varied between a few minutes (e.g., 15 minutes after the Webinar) and several days (e.g., five days). We included this information in the method section and its limitations.

  

- Discussion: authors must make a real effort to present the practical applications of their work. Outside of the pandemic, what can we learn from this work?

Answer 5: In the discussion, we have argued the implications that could help to study the levels of physical activity of students and advance in the promotion of physical activity and the fight against a sedentary lifestyle in the university.

Specific comments:

- Abstract: The wording of the objective is confusing. It is not the effect of the pandemic on pre-lockdown and lockdown that is being explored but the effect of active transportation, uninterrupted sitting time, and screen study time on physical activity and sitting time during the lockdown.

Answer 1: We modified the objective in the abstract as it’s suggested, thanks for pointed it out.

  

- Introduction: I don’t understand what hypothesis 3 is based on. The increase in online courses during confinement is a phenomenon independent of the screen time prior to confinement.

Answer 2: Hypothesis 3 is linked to screen time to study. University students use screens to study (i.e., computers). The computer use is also a prevalent behavior in this population. Therefore, the explosion of this type of screen use increased during Covid. We included new references to present this idea. Please see Castro et al., 2021 reference).

  

- Measures – Physical activity and Sitting time: Be careful of inconsistency. A sentence mentions that the data of the last seven days are requested while in the example it is mentioned “prior to confinement” and “during the confinement”.

Answer 3: You are right; there was an inconsistency in these phrases. We did not see them when we created the online questionnaire, as we used the IPAQ statement. We should have modified this sentence. We have now modified it so that this element will be considered in other studies.

  

- Measures: Some data were measured only on weekdays while others are measured in general. Why sitting time, uninterrupted sitting time, screen time, and screen time studying data were not measured separately for weekdays and weekends? We might hypothesize there are differences.

Answer 4: we considered only week days because we measured the university context or time when students were “at university”, having lectures. Another idea was to measure physical activity with a validated questionnaire (IPAQ); for that reason, the measure was a general one. We modified the sentences to improve the questionnaires’  consistency in case other projects want to assess the same behaviors.

  

- Statistical analysis: Why sex and age were included in the regression model as control variables? You need to justify this in the introduction part.

Answer 5: We included the sentences to justify the inclusion and control of these variables in our models. Please see the reference Motevalli et al., 2023.

  

- Statistical analyses: the 4th hypothesis is not mentioned.

Answer 6: We mentioned the four models which allowed us to test our fourth hypothesis. Please see this modification in statistical analysis.

  

- Results: “Students spent more than 10 hours per day using screens, of which approximately five hours were for …”. Please specify “during confinement” if this data refers to this period.

Answer 7: We specified that the data was during confinement.

  

- Table 1: The data in the right-hand column is based on headcount, not frequency.

Answer 8: We changed frequency by “ *n” *on Table 1.

  

- Hypothesis: Please reformulate “Multiple regression models showed that when there was a negative effect in changes, it opposes the effect of confinement, if there is a positive change effect, it increases the impact of confinement.” for the lecturer.

Answer 9: We reformulated the phrase you indicated. 

  

- Tables 2 and 3: Does “habitual” corresponds to data during confinement? This term is not used in the manuscript.

Answer 10: “Habitual” corresponds to data in “normal periods” without confinement. We include this information in the table 2 and 3.

  

- Results – H3: Is this the screen time to study **before confinement** that predicts a lower increase in screen time during confinement?

Answer 11: It’s the screen time to study before confinement. It was modified in the H3.

# Reviewer report: Reviewer response for version 1

**DOI:** 10.5256/f1000research.129683.r150195

**Published:** 2022-10-11

**Authors:** Akhmad Habibi

**Affiliations:** 1 Universitas Jambi, Jambi, Indonesia

The paper has the potential to contribute to the related fields. However, some improvements should be considered:

1. Lit. review related to the four hypotheses should be supported by prior studies.
*a.      H1: The first hypothesis was that the longer the duration in minutes of students’ active transportation trips prior to confinement, the greater the reduction in their physical activity levels, excluding active transportation time during confinement.*
* b.      H2: The second hypothesis was that the greater the uninterrupted sitting time prior to confinement in students, the greater the increase in the amount of time they would spend sitting during confinement.*
* c.      H3: The third hypothesis was for screen time; due to the increase in online courses during confinement, the longer the screen time studying prior to confinement in students, the greater the increase in their screen time during confinement.*
* d.      H4: Finally, we hypothesized that there would be no relationship between active transportation, sitting time and screen time during confinement.*
  It is essential to integrate previous work discussed and critiqued in the literature review and discussion sections with the findings and tone of the study and bring out what was done in this study which addressed some of the shortcomings of those studies.
2. The gap between this research and prior research should also be more detailed. Besides, the significance of the current study is recommended to be more clear and more novel. In this sentence, “ *Most studies about confinement in the COVID-19 pandemic have focused on total times of changes in physical activity levels, sitting time, and screen time. For this reason, the main objective of this study was to assess if active transportation time, uninterrupted sitting time, and screen time spent studying prior to confinement were factors influencing these changes*”. The studies are not cited and elaborated on. I suggest that the authors elaborate more on them, highlights on each finding of the works should be informed.
3. With N=2873 samples involved in the study, the authors need to conduct normality tests like Q-Q plot, skewness, and Kurtosis. The normality test is used to determine whether sample data has been drawn from a normally distributed population (within some tolerances). Without this, bias could be high for further statistical analysis.
4. The conclusion could be improved to motivate the related communities to get down to actionable, practical, and academic implications. The authors missed the limitation, implications, and recommendations for practical and theoretical sections. Please include those parts in the conclusion of the study.

Is the work clearly and accurately presented and does it cite the current literature?

Yes

If applicable, is the statistical analysis and its interpretation appropriate?

Partly

Are all the source data underlying the results available to ensure full reproducibility?

Yes

Is the study design appropriate and is the work technically sound?

Partly

Are the conclusions drawn adequately supported by the results?

Partly

Are sufficient details of methods and analysis provided to allow replication by others?

Yes

Reviewer Expertise:

Statistics, educational technology

I confirm that I have read this submission and believe that I have an appropriate level of expertise to confirm that it is of an acceptable scientific standard, however I have significant reservations, as outlined above.

# Response

**Published:** 2023-11-28

**Authors:** Gonzalo Marchant

**Affiliations:** University of Rouen Normandy, France

Dear Colleague,

 First of all, we would like to thank you for your comments and suggestions.

 Secondly, you will find our detailed responses to your comments in the following lines.

 Thus, you will see your comments followed by our answers, including the references that allowed us to complete our arguments.  

1. Lit. review related to the four hypotheses should be supported by prior studies.

**Answer 1:** As requested, we have further developed our propositions, including new references in the introduction section, to complete the arguments according to our hypotheses.

*a.      H1: The first hypothesis was that the longer the duration in minutes of students’ active transportation trips prior to confinement, the greater the reduction in their physical activity levels, excluding active transportation time during confinement.*

**Answer 1.a: **We justified the first hypothesis with the following arguments: Students who use active transportation are more active than those who do not. Among those who do, those who take longer trips also have a higher total physical activity time. Consequently, confinements reduce the active transportation to the university, thus reducing the total physical activity of the more active students during this period. Please see references in the first paragraph: Motevalli et al., 2023; Meyer et al., 2020. The second paragraph includes Yang et al., 2012 reference to complete our arguments according to the first hypothesis.

*b.      H2: The second hypothesis was that the greater the uninterrupted sitting time prior to confinement in students, the greater the increase in the amount of time they would spend sitting during confinement.*

*c.      H3: The third hypothesis was for screen time; due to the increase in online courses during confinement, the longer the screen time studying prior to confinement in students, the greater the increase in their screen time during confinement.*

**Answer 1.b and 1.c: **For the second and third hypotheses, we have given two arguments to improve the theoretical framework that allows us to justify them. Thus, we considered that having online courses during confinement increases students' time spent sitting in front of a screen (Please see Castro et al., 2021 reference). The second argument considers the literature showing that screens increase sitting time as it is uninterrupted and longer. Please see the reference Castro et al., 2021.

*d.      H4: Finally, we hypothesized that there would be no relationship between active transportation, sitting time and screen time during confinement.*

**Answer 1.d: **Regarding the fourth hypothesis; the new version includes arguments explaining why we think there are no relationships between active transport, sitting time, and screen time. Please see references Heller et al., 2023; Trott et al., 2022. 

 It is essential to integrate previous work discussed and critiqued in the literature review and discussion sections with the findings and tone of the study and bring out what was done in this study, which addressed some of the shortcomings of those studies.

**Answer 1**.d1: We have included this information in the literature review and present the elements that allow us to advance knowledge about the levels of physical activity and sedentary behaviors in university students.

**Answer 1.d1 bis:** The literature review section includes new articles and analyses that allow us to highlight elements missing from previous studies. The main argument has to do with the type of variables included (e.g., duration of active transport journeys and uninterrupted sedentary time, duration of time spent using screens to study) in this study and the behaviours assessed (active transport, sedentary behavior, and screen use).

 2. The gap between this research and prior research should also be more detailed. Besides, the significance of the current study is recommended to be more clear and more novel. In this sentence, “ *Most studies about confinement in the COVID-19 pandemic have focused on total times of changes in physical activity levels, sitting time, and screen time. For this reason, the main objective of this study was to assess if active transportation time, uninterrupted sitting time, and screen time spent studying prior to confinement were factors influencing these changes*”. The studies are not cited and elaborated on. I suggest that the authors elaborate more on them, highlights on each finding of the works should be informed.

**Answer 2:**

 We have detailed the arguments that allow us to show the gap between this research and previous ones. In addition, we have added new literature to elaborate these arguments in a more complete, precise and coherent way. Consequently, we have modified the sentences and content you have indicated.

 3. With N=2873 samples involved in the study, the authors need to conduct normality tests like Q-Q plot, skewness, and Kurtosis. The normality test is used to determine whether sample data has been drawn from a normally distributed population (within some tolerances). Without this, bias could be high for further statistical analysis.

**Answer 3:**

 We are aware that our independent variables do not follow a normal distribution. Thank you for this comment. However, two arguments allowed us to carry out the multiple regression analyses.

 Our sample size is 2873 students. According to the Central Limit Theorem, as sample sizes get larger, the distribution of the means approaches a bell curve even when the underlying distribution is highly skewed (Fisher, 2011; Sainani, 2012). Consequently, biases could be important in small samples (≤ N= 80). Thus, for our sample, linear regression provides a convenient and practical alternative (Lumley et al., 2002).

 The second argument concerns physical activity behaviors. For example, data about physical activity is rarely bell-shaped. Instead, these follow a right-skewed distribution: they have a cluster of values at zero or zero-inflated distributions (non-practitioners for moderate-to-vigorous physical activity), another bunch in the low-to-moderate range, and a few extreme values to the right (highly active).

 For more information, see:

 - Fischer, Hans (2011). A History of the Central Limit Theorem: From Classical to Modern Probability Theory (PDF). Sources and Studies in the History of Mathematics and Physical Sciences. New York: Springer. doi:10.1007/978-0-387-87857-7.

 - Lumley T, Diehr P, Emerson S, Chen L. The importance of the normality assumption in large public health data sets. Annu Rev Public Health. 2002;23:151-69. doi: 10.1146/annurev.publhealth.23.100901.140546.

 - Sainani KL. Dealing with non-normal data. PM R. 2012 Dec;4(12):1001-5. doi: 10.1016/j.pmrj.2012.10.013.

 4. The conclusion could be improved to motivate the related communities to get down to actionable, practical, and academic implications. The authors missed the limitation, implications, and recommendations for practical and theoretical sections. Please include those parts in the conclusion of the study.

**Answer 4 :**

 We included the limitations and proposed practical and theoretical recommendations about our results in the study's conclusion.
